# Community partner perspectives on the implementation of a novel safer supply program in Canada: a qualitative study of the MySafe Project

**DOI:** 10.1186/s12954-023-00789-8

**Published:** 2023-04-28

**Authors:** Manal Mansoor, Annie Foreman-Mackey, Andrew Ivsins, Geoff Bardwell

**Affiliations:** 1 British Columbia Centre on Substance Use, 400-1045 Howe Street, Vancouver, BC V6Z 2A9 Canada; 2 Department of Medicine, St. Paul’s Hospital, University of British Columbia, 608-1081 Burrard Street, Vancouver, BC V6Z 1Y6 Canada; 3 School of Public Health Sciences, University of Waterloo, 200 University Ave. West, Waterloo, ON N2L 3G1 Canada

**Keywords:** Safer supply, Qualitative research, Canada, Community partner perspectives, Implementation science, MySafe Project, Overdose crisis

## Abstract

**Background:**

The adulteration of the illicit drug supply with fentanyl and its analogues is driving the ongoing overdose crisis in North America. While various harm reduction interventions address overdose-related risks, there is growing interest in safer supply programs, including the MySafe Project which utilizes a biometric dispensing machine that provides pharmaceutical opioid alternatives to the toxic drug supply. However, the experiences and perspectives of professional community partners on program implementation remain unexplored. This study aims to examine professional community partner perspectives on the feasibility, as well as barriers and facilitators to the implementation of the MySafe program.

**Methods:**

Semi-structured qualitative interviews were conducted with 17 professional community partners involved in program implementation across four pilot locations in Canada. Thematic analysis of interviews focused on perspectives on safer supply, barriers and facilitators faced during program implementation, and recommendations to inform future scale-up of low-barrier safer supply models across Canada.

**Results:**

Participants identified a variety of barriers, including the dependence on clinician buy-in, coupled with regulatory and logistical constraints. In addition, some participants perceived hydromorphone to be an inadequate substitute to the increasingly toxic street opioid supply. Lastly, technical difficulties were described as barriers to service uptake and delivery. Conversely, having political and community buy-in, availability of wrap-around services, and collaborative communication from the MySafe team served as facilitators to program implementation. Though community partners preferred establishing MySafe machines into existing community organizations, they also discussed benefits of housing-based MySafe programs. The potential role of this program in mid-sized to rural cities was also emphasized.

**Conclusions:**

To address the overdose crisis, there is an urgent need to implement and evaluate novel solutions that address supply drivers of crisis. Community partner-informed research plays an integral role in ensuring program acceptability and proper implementation. Our findings identify current gaps and facilitators underlying the efficacy of one such model, together with future directions for improvement. Participant recommendations included a diversification of medications offered and types of locations for MySafe programs, a streamlined national approach to prescribing guidelines coupled with more robust training for healthcare professionals, and an emphasis on service delivery within an integrated services model. Our findings underscore a potential gap between the goals of healthcare providers in ensuring comprehensive care and the necessity for low-barrier models such as MySafe that can function both within and outside of integrated service models.

## Background

Canada is grappling with an ongoing overdose crisis driven by an illicit drug supply of both increasing potency and variance, greatly exacerbating drug use-related risks and harms to people who use drugs (PWUD) [1, 2]. A range of public health measures have sought to target these unintentional deaths, including: naloxone programs; supervised consumption services (SCS); oral and injectable opioid agonist treatment (OAT); and safer supply programs [3–8]. Safer supply is defined as the provision of a pharmaceutical (i.e., unadulterated) alternative of known quantity and quality as a means to decrease reliance on the toxic illicit drug supply to reduce overdose risk to PWUD [9]. This intervention extends the concept of existing OAT programs by providing access to pharmaceutical-grade opioids to PWUD regardless of their level of engagement in treatment [3]. Furthermore, while traditional OAT programs have demonstrated effectiveness in reducing all-cause overdose mortality risk [10, 11], safer supply presents a lower barrier avenue for substance use care for PWUD whose needs are not being met by existing OAT options, or those who may not be interested in receiving treatment [9].

Following the promising outcomes of randomized controlled trials demonstrating the effectiveness of injectable diacetylmorphine and hydromorphone in Canada [12, 13], injectable hydromorphone was approved for the treatment of opioid use disorder within Canada [14]. Meanwhile, clinical trials on the use of diacetylmorphine for the treatment of opioid use disorder have shown positive health benefits, including several European countries and at one clinical location in Canada [6, 12, 15–17]. However, the use of diacetylmorphine is restricted to opioid use disorder treatment and has not yet expanded as a safer supply option [18]. Since then, off-label hydromorphone has been made clinically available on an individual basis to PWUD considered at high risk of overdose by their physicians as well as via community-based tablet distribution programs in select Canadian cities, including Vancouver [3, 19, 20]. Ivsins et al. [3] highlighted the impacts a low-barrier hydromorphone tablet distribution program in Vancouver, noting that not only were participants’ illicit drug use and overdose risk reduced, but other measures of well-being also improved through the program (e.g., improvements in economic, overall health and well-being, and pain management needs). With the onset of the COVID-19 pandemic, access to safer supply has been expanded within British Columbia through the implementation of the Risk Mitigation Guidelines permitting the prescription of select opioids and other controlled substances to individuals in order to encourage physical distancing and reduce overdose risk due to supply disruptions owing to the pandemic [21]. While these measures represent encouraging developments toward a robust national response to the overdose crisis, there is minimal research evaluating these novel interventions [22].

Existing safer supply literature primarily accounts for the perspectives of PWUD enrolled in safer supply programs in Canada, which is integral for ensuring relevancy and acceptability of public health measures. While there has been a recent study capturing professional community partner perspectives on safer supply in the context of COVID-19 pandemic [23], no study to date has focused on systems-level determinants of safer supply implementation nor has there been any study focusing these perspectives as they relate to the MySafe program (the focus of this article). The alignment of professional community partner expectations in the design and implementation of novel public health measures and programming is critical to ensuring relevance to the target population and smooth program roll-out. This is due to the high degree of cross-collaboration across federal and provincial governments, healthcare providers, and community partners, among others [24, 25]. The integration of research and practice in ensuring equitable service delivery and uptake can be attained through meaningful researcher-community partner collaborations where professional community partners are involved in health systems change planning at all stages of program roll-out [26–28]. Past iterations of collaborative working models between researchers and community partners have shown considerable success in terms of program relevancy and subsequent uptake, demonstrating the importance of community partner-informed research [26, 29, 30].

In December 2021, the MySafe program was piloted across Canada as a means of providing a low-barrier, regulated, and safer supply of opioids as a response to the increasingly adulterated and inconsistent illicit drug supply. The first of its kind, this biometric machine dispenses tablet hydromorphone on a pre-determined schedule, with machines currently located within either housing-based or community organization-based settings (i.e., SCS) [31]. The use of these medications is non-witnessed, and participants have the choice to administer them intravenously, intranasally, or orally. Participants in the program are eligible for enrollment if they have a history of overdose, are regularly using opioids, and have had fentanyl detected in their urine samples. In this study, we sought to examine the feasibility and implementation of the MySafe program among professional community partners across Canada.

## Methods

This study employed a qualitative approach and was conducted as part of a larger evaluation of the MySafe program. Our study draws on semi-structured interviews with key professional community partners (*n* = 17) involved in the design, proposal, and implementation of the program. Professional community partners included clinicians (i.e., physicians, nurses, pharmacists) (*n* = 7), program managers and executive directors (*n* = 7), and political and health authority representatives (*n* = 3). Interviews focused on perspectives on safe supply, barriers and facilitators faced during program implementation, as well as recommendations to inform the future scale-up of the MySafe Program specifically and low-barrier safer supply models more generally across Canada. At the time of data collection, only one of the four pilot locations was operational, while the others were in early implementation stages.

Recruitment was purposive and done primarily through MySafe program staff members at each of the initial implementation sites: (Vancouver (*n* = 6) and Victoria (*n* = 4), British Columbia; London, Ontario (*n* = 2); and Dartmouth, Nova Scotia (*n* = 5). AFM and GB outlined study objectives and eligibility criteria via an enrollment script sent to the MySafe program staff, who then forwarded this email to a list of relevant parties that played key roles within program implementation based on their expertise and degree of involvement across all the aforementioned sites. The list included community partners that were directly involved in day-to-day operations (i.e., clinicians, program managers) as well those who were positioned to enact systems-level change in the larger context within which such safer supply models exist (i.e., provincial regulatory bodies, health authorities). Participants self-enrolled in the study by contacting the study team directly. Participation in this evaluation was voluntary, and a research team member was available to answer any queries prior to study enrolment. Participation in this evaluation was voluntary, and a research team member was available to answer any queries prior to study enrolment. All participants provided written, informed consent prior to the commencement of each interview, and their data were anonymized upon collection. In addition, due to the small sample size of this study, participant locations are not specified for any quotes included within this publication in an effort to maintain confidentiality.

Data were collected between June and September 2021 in the form of semi-structured interviews conducted remotely via phone or Zoom. These one-on-one interviews typically lasted 30–60 min and were facilitated by an interview guide. Interviews were audio-recorded and transcribed verbatim by externally contracted professional transcriptionists. An initial coding framework was developed by AFM and GB to guide the identification of themes relevant to a priori categories within the interview guide. Transcripts were imported into and coded with NVivo 12, a qualitative data management and analysis software program. Applied thematic analysis [32] was guided by the study objectives, namely (1) an exploration of community partner narratives around the factors contributing to the success of program implementation, (2) identification of potential barriers to program roll-out and (3) recommendations to inform the future application and scale-up of similar low-barrier safer supply models within Canada. In order to extract data pertinent to the a priori categories within the interview guide, interview transcripts were initially reviewed using a line-by-line deductive approach and later refined with an iterative, inductive approach to represent additional themes identified by the research team [33]. Ethical approval was obtained through the University of British Columbia/Providence Health Care Research Ethics Board (H21-01413).

## Results

### Barriers to implementation

#### Clinician buy-in

Given the broader policy context of a lack of a streamlined national approach to the application of prescribing guidelines within Canada, the implementation of the MySafe program was driven largely by professional community partner buy-in and their sustained engagement, especially that of clinicians. There was a broad consensus among study participants that continued reliance on clinician support for program uptake presented a significant barrier to its implementation. Participants identified several concerns with the overreliance on clinicians. Firstly, as prescribing guidelines are left open to interpretation by the regulatory Colleges, program access hinges on individual prescribers’ attitudes toward safer supply and harm reduction. Conservative understandings of the guidelines restricted equitable access of safer supply medications to PWUD in certain jurisdictions. According to one program manager:

*I think the idea of the machine is great in terms of like accessibility obviously and like if we didn’t have to associate a prescription to the machine, then it’s just a great delivery method where people can access a safer supply… it’s limited because we have to have a physician there to the machine to get a hold of those medications… So I think that there’s a ton of potential there and [it] …is only limited by… criminalization, prohibition, and... not having access to drugs without going through a physician* (S14—program manager)

Secondly, some participants who were onboarding pharmacies for the provision of safer supply medications for MySafe machines were met with reluctance and fear of collaborating with a safer supply program. Some participants noted that this was due to the MySafe program “*not [being] set up to be as good financially for the pharmacies”* (S9—program lead). It was also reported that prescribers wanted to avoid reproachment from their respective regulatory Colleges that may impact their licensing ability if they engaged with the program. This led to hesitation among clinicians and pushback from the association of pharmacies in some jurisdictions as this program was perceived as diminishing their reach, as illustrated in the following quote:

*I think the pharmacies are interested in the technology but they’re battling with the fact that they don’t want to be sanctioned by the Colleges either and the Colleges don’t want these machines there. The other pushback from the association of pharmacies is they have a lot of concerns that these machines will replace the pharmacist and things, so that’s kind of an existential threat that MySafe obviously doesn’t, MySafe isn’t going to do that* (S9—program lead)

Lastly, some health authority participants felt that the MySafe model disconnects program participants from holistic and integrated healthcare approaches. Given that many PWUD live with co-morbidities, several professional community partners preferred the pharmacy or community-based health services model because there was concern that program participants may begin prioritizing their safer supply medications over those for other illnesses if the machines were placed in non-clinic-based settings. By isolating the machines in such settings, there was concern that it would disrupt existing connections with care providers and silo program participants away from other available health and social supports. One participant explained:

*Some of the challenges I think are that it disconnects the access to that medication or product from the rest of the person… a lot of people are on various medications, daily [dispensed] or otherwise… so, it becomes sort of less clear, …what the advantage is to separating that… from going to the pharmacy because there would be concern that people would maybe, “Oh, I’ll just go to that machine and just get my Dilaudids [i.e., hydromorphone] and I’ll ignore my other medications that are at the pharmacy,” or vice versa* (S6—health authority)

As a result, many participants who were health authorities felt that the machines should be integrated into well-established community-based pharmacies or harm reduction organizations for maximal benefit.

#### Logistical and regulatory constraints

Varying levels of policy challenges were encountered by a number of participating sites, who at the time of data collection, had yet to successfully implement the MySafe program. In one location, regulatory restrictions around the dispensing and storage of controlled substances in non-pharmacy-based settings impeded program roll-out:

*There’s regulatory challenges with it [MySafe implementation] because the College of Pharmacies is really particular around dispensing and holding of medication, as they should be, but it’s created some challenges…We worked with Dispension [i.e., the dispensing machine company] for a few months, worked with our partners at [the pharmacy], made it really clear that we couldn’t, we don’t have the policies or the proper structures in place to house narcotics… So then we found ourselves with a barrier.* (S15—program lead)

Furthermore, having regulatory, political and clinician support was at times insufficient if there were on-site logistical constrains such as reluctance on part of property owners or management to house the machines. Private property owners that were not acquainted with harm reduction approaches perceived these machines as normalizing and promoting substance use and thus pushed back against such initiatives. These structural barriers limited the reach of MySafe programs in residential settings:

*But as soon as it came to the actual owner of the hotel, it was immediately shot down. They don’t want to promote drug use in the sites. Just pure ignorance.* (S12—clinician)

Several participants also felt that while the current model may apply well in certain settings (i.e., large urban areas), it failed to account for the unique needs and constraints of others (i.e., small to mid-sized cities). Large urban cities such as Vancouver were perceived to be “*very forward-thinking”* (S5—program lead) by professional community partners from smaller cities when comparing their respective local contexts. Participants felt that implementation of MySafe was not going to be effective if done in the same manner in all locations without accounting for the unique local and provincial contexts. For example:

*Barrier is lack of understanding for the context, right? Like just thinking that because it works one way in B.C., that it’s going to work that way everywhere else. And I mean, it just isn’t… I feel like every meeting I have, I have to explain the provincial context, and explain why it’s the best position here. And so, I feel like I’m constantly having the same conversation, time after time after time* (S5—program lead)

Instead, tailored implementation specific to each community’s needs was seen as essential for feasible and successful roll-out of the program.

#### Accessibility and adequacy

While the MySafe program is intended as a low-barrier program with *“very loose”* eligibility criteria (S9—MySafe lead/clinician), access to the program was limited by differences in enrollment criteria between sites. In one location, there was reportedly pushback in enrolling those with certain comorbidities such as liver or kidney conditions whereas this exclusion criteria did not apply for other forms of opioid agonist therapy (OAT; e.g., methadone). Participants from another location reported that PWUD who were already accessing OAT were ineligible for MySafe program enrolment, as illustrated by the following excerpt:

*This is what we encountered when safer supply first started and the Colleges were telling people … [that] because they’re already on OAT so why would they need Dilaudid for safe supply? But again, …take your therapeutic hat off, put your harm reduction hat on. He’s already using off the street. We already have heroin testing positive in his urine so OAT is not working well enough for him or her. So why would we say to them “No, keep on using dirty street drugs.” It doesn’t make any sense. This is a person who’s already engaging in care with you… saying “Hey, I want to try and get off my stuff but I’m having a hard time”… We should be saying “Do you need this in addition to your OAT?”* (S11—clinician)

In addition, hydromorphone was considered by some to be an inadequate substitute to the increasingly toxic unregulated drug supply. As noted by a study participant: *“what’s in it should be dictated by what is being requested”* (S8—program manager)*.* Another participant explained further:

*If this was 2015 and just at the beginning of the emergence of fentanyl in the drug supply, the MySafe machine would probably have been this amazing tool that we could use. And now, however, we’re 6 years later and people’s tolerances are so high that they’re not really being met by tablet safer supply and there isn’t going to be a doctor in the world that’s going to prescribe injectable solutions via a vending machine.* (S16—clinician)

As a result, numerous participants called for a diversification of medication offerings in the MySafe program to ensure relevancy and perceived it as a facilitator to program engagement.

#### Technical issues

During initial implementation, frequent technical difficulties “*ranging from software updates that never happened to mechanical jamming of the medication cartridges in the vending machine*” (S16—clinician) were identified by many participants as a critical challenge. Professional community partners from an operational MySafe site reported frustration among program participants due to persistent technical problems to the point that some opted to re-enroll in pharmacy-based programs. Technical support was provided by the dispensing machine company; however, support tended to be limited by differences in time zones. For example:

*There was one day where a dose got stuck in the machine. And nobody on site had a key to open it. I think they had to contact the pharmacy and the pharmacy was busy so they had to contact the nurse who did the deliveries and she was on outreach… you don’t want to have everybody being able to open the machine up but you need to have access for things like that… I mean there have been a number of software glitches, with difficulties registering, difficulties with affecting the biometrics…* (S12—clinician)

Participants pointed to the shortcomings of having medication dispensation that was mediated by technology as opposed to staff relied heavily on the software working reliably each time as a barrier.

### Facilitators to implementation

#### Community/political buy-in

To ensure smooth implementation of the MySafe program, it was reported that having political and community buy-in alongside adequate funding was instrumental especially during the initial phases of setting up the program. Having “*community willingness to work outside the box”* (S13—program manager) to incorporate novel and innovative mechanisms for healthcare delivery was considered an essential prerequisite at different stages of program roll-out. In this regard, larger urban centers experienced a smoother and easier process integrating MySafe programs as there was a greater understanding of harm reduction and a higher willingness and ability to fund such initiatives. For example:

*Several pharmacies [were] very willing and very helpful… partly because they were already involved. Like they already were working with one or two other programs around doing this type of work, so I think… it was easier in a bigger city where there’s pharmacists who are already doing addictions type work and are pretty familiar with the substances*. (S1—program lead)

Conversely, relatively smaller urban settings tended to have scarcer resources, fewer prescribers, and more conservative approaches to substance use. As a result, professional community partners from these areas highlighted the varying levels of structural barriers encountered when trying to introduce MySafe programming into their existing continuum of care. According to this participant from a smaller city:

*It’s very controversial, right? Like a lot of people, like the Health Authority… they just had this idea that people just walk up to the machine and put their hand out… there was no involvement of a physician… because that’s the way it was like highlighted in the media… But that’s not what it is… I’ve had to explain I don’t know how many times, like this is still a medical model… people still have to be assessed by a doctor. They’re still going to have to do a certain amount of urine drug screening. You know, they’re still going to have to have contact with people… so that has been a downside of it, is because especially in a province that doesn’t have safer supply, to then come out with this big hoopla machine* (S5—program lead)

The political will to fund safer supply initiatives coupled with support from local prescribers and property management to house MySafe machines played a key role in determining program roll-out.

#### Availability of wrap-around services

Participants identified access to additional wrap-around services as a key facilitator to implementation and felt that it bolstered program uptake and efficacy. Embedding MySafe machines in established community organizations such as SCS, community clinics, or other harm reduction services would provide program participants with further linkages to support along the continuum of care. Many professional community partners felt that the availability of such supports in close proximity of the machine would have a positive cascading impact on other key determinants of health such as housing and employment, as well as direct impacts on drug use-related risks. As one participant explained:

*As far as I understand the proposed kind of model of care might look a little different in each city but would generally involve the peer support workers and nursing support and then prescribers and case manager and that kind of thing* (S7—clinician)

Furthermore, there was broad sentiment that having program participants frequent MySafe sites with wrap-around services available daily would allow for greater rapport between staff and program participants promoting more positive health outcomes. For example:

*We didn’t ever expect the [funding] to come through, so I think having that has been awesome… So I think when we [were] talking it up with neighbours and the city and stuff, like it was really like, “Hey, like this is providing this service but it’s not alone.” I think most people’s fear was we just got a vending machine… and be isolated but I think we’re pointing out, hey, like we got a lot of staff time associated with this and actually give staff more time to do the work that they ought to be doing, which is having the conversations and all that. So, I think that that tie-in is massive* (S1—program lead)

Having MySafe machines nested within existing community organizations with pre-established wrap-around services would enable professional community partners to secure funding and support from physicians and regulatory bodies as it alleviated concerns around the MySafe model deviating from holistic healthcare approaches.

#### Responsive communication

Several professional community partners from operational sites highlighted a high degree of responsiveness to troubleshooting requests, resource sharing, and access to technical support resources that allowed for a seamless collaboration between site staff and the MySafe Society. As discussed previously, frequent technical difficulties were reported with the machines at an operational site; however, these challenges were mitigated with responsive communication with the MySafe team. It was noted by participants that the MySafe Society conducted frequent check-ins, including site visits, and provided opportunities for information sharing between staff at different MySafe locations. Their support resources were also considered concise and relevant which allowed for community partners such as prescribers and site staff to understand and incorporate the program into their practices with ease. These streamlined efforts contributed to a conducive environment for program implementation and were considered a key strength of the MySafe model. According to one participant:

*The operations manual and like all of the instruction like the support that has come from MySafe Society has been great… when we sent the manual to our physician initially who agreed to prescribe, like it was all very straightforward to him and he didn’t have any concerns about it… I think because there were resources available to them, like here’s the instruction manual and… here’s a pharmacist you can call in Vancouver who can talk to you about their experience with it.* (S14—program manager)

These resources and efforts served to mitigate some of the technical difficulties encountered as well as to help secure the buy-in of clinicians—a necessary prerequisite for program enrollment.

### Perspectives on implementation settings

Aside from describing implementation barriers and facilitators, participants also discussed their perspectives on what types of settings would benefit from a program like MySafe. For example, they highlighted that having not one but several machines across a variety of settings would be a salient determinant of program uptake. In order to ensure acceptability across different contexts, study participants emphasized that program locations should be determined on an individual basis with site-specific professional community partners. However, there was a general preference to embed MySafe machines into trusted services that already exist in communities (e.g., SCS, pharmacies), both as a means of securing political and community buy-in as well as providing wrap-around services to promote holistic approaches to the healthcare of PWUD who often live with comorbidities. This would also help bypass policy barriers faced by housing providers regarding onsite program implementation.

Both SCS and pharmacy-based settings had similar benefits in that they had existing managerial staff and security, and established ties with the target community. However, both these settings lacked the ability to offer 24-h access to medications and were not accessible to those with transportation or geographical constraints, which were identified as critical barriers to access for PWUD. In addition, professional community partners reported that pharmacy-based settings may potentially negate the low-barrier aspect of the MySafe model due to stigma attached with medicalized settings. According to one participant:

*…if you have it in the pharmacy then you might as well just write the order as daily dispense and then the pharmacist can talk to the patient and have that conversation and build that rapport…The only advantage that I can see [of] having it in a pharmacy is that we already have pretty significant security because of what we store…Most pharmacies aren’t open 24 hours. And again, we’re dealing with clients who might be out all night partying and doing stuff and then they sleep during the day and they don’t get up until 5:00 at night so how is that gonna be helpful if you’re putting into a pharmacy? Even if they say close at 9:00 pm which is pretty late in my mind. That might be pretty early for my patients.* (S11—clinician)

Housing-based settings addressed some of these challenges, namely the provision of unrestricted 24-h access, safe storage within residence, and involved no travel for the people that lived there. However, the transient nature of people staying in supportive housing-based settings raised concerns about what access would look like in the event of an eviction or a move-out. Additionally, some participants argued that such a model disconnected program participants from other supports and medications that could be accessed from clinical settings. Lastly, many community partners considered the potential of this program in small to mid-sized cities as especially impactful due to the ability to scale up cost-effectively in places with limited resources and support. Having even one machine *“allows one doctor to support 40 or 50 people”* (S8—program manager), thereby allowing for areas with low prescriber availability or buy-in to have avenues for safer supply access. However, geographical restrictions may hamper proper implementation in rural settings due to the dispersed distribution of PWUD across vast distances [34]. As one professional community partner stated:

*Unless you have agencies that are able to invest in like, you know, 10 machines to cover the region, right? So it works, and it would work in—a mid-sized city for the folks who reside in the core or work in the Downtown Eastside…but if I think about like in Ontario, where [the] highest fatal overdose rates are, which is like Timmins and Porcupine, Thunder Bay, Sudbury, people are spread out at a very wide area. So one machine, which is typically, I think, what most agencies could hope to afford at this time, is just not going to do it.* (S17—clinician)

While the program holds potential for scale-up in mid-sized to rural cities, funding challenges and political was reported as a potential barrier within these settings.

## Discussion

This qualitative study examined the perspectives of professional community partners on the feasibility of, and barriers and facilitators to, the implementation of the MySafe program across four initial pilot locations in Canada. Our findings highlighted several barriers to program roll-out: the continued reliance on clinician buy-in for program uptake, provincial and regional restrictions resulting in inequitable program access, and the perceived inadequacy of hydromorphone to meet the needs of PWUD coupled with technical difficulties with the machine. Conversely, professional community partners identified several key facilitators, including: the provision of wrap-around services alongside a MySafe machine, securing political and community support of the program, and having a collaborative and communicative working partnership between local community partners and the MySafe Society. In light of these identified barriers, participants shared several recommendations for improvement, emphasizing consultation with local community partners to promote program relevancy to their individual contexts. Moreover, embedding machines within trusted community organizations and diversifying medication offerings and program locations to include housing-based settings were proposed as possible improvements to service delivery.

Our study findings speak to the importance of community partner involvement in program design and implementation given that plans for two of the four pilot sites included in this study did not materialize. As of August 2022, implementation went forward in Vancouver and Victoria, British Columbia, whereas the program never became operational in London, Ontario and Dartmouth, Nova Scotia, reiterating participant narratives around greater ease of implementation in large, urban cities compared to smaller urban settings. Past implementation science research has supported the role of context in determining program impact, advocating against a one-size-fits all approach in the development of public health interventions [35–40]. The attitudes of community partners have a key impact in shaping the socio-cultural environment in core areas of public services (e.g., policing, healthcare, courts) and affect the provision of care by allocating funds in ways that deprioritize treatment needs, commonly observed in less urban areas [41–43]. Urban–rural disparities are rampant within Canada, with more liberal and experimental overdose crisis responses explored in urban areas such as Vancouver, whereas less urban to rural areas experience greater stigma, significantly lower linkages to care, and lesser availability of OAT prescribers in general [43–45]. As a result, professional community partners highlighted the merits of a tailored approach to implementation which accounted for the unique contextual constraints that defined their communities, without which attaining the buy-in of clinicians and governing bodies became challenging. The inclusion of professional community partners in pre-implementation stages lends a deeper understanding of the barriers faced by existing service frameworks and the cultural perspectives that guide them. This study adds to the repertoire of the literature that explores community partner perspectives on the implementation of various harm reduction services including fentanyl drug checking and SCS; however, it remains one of the only few studies reporting on perspectives on safer supply [5, 46–48]. The understanding of these limitations may then serve as guiding principles in the development of best practices in a particular context. While the inclusion of PWUD in the design, implementation, and operation of public health interventions has been extensively studied and considered best practice [48–52], the role of professional community partners is crucial to ensuring relevancy and acceptability within the local context in question. Consistent with the past literature, harm reduction programs benefit from community partner-informed research by being better positioned to address issues of program sustainability and uptake due to the impact of professional community partner opinion on public policy [46, 53–56].

Study participants showed a strong preference for the MySafe machines to be embedded within other community supports as a way to connect hard-to-reach populations to a continuum of care (e.g., OAT, medical care for co-morbidities, housing and employment supports). The existing literature attests to the benefits of integrated service models in engaging PWUD in overall health-promoting behavior and securing community support [57, 58]. For example, a recent qualitative study examined professional community partner recommendations for service uptake among youth who use drugs and reported wrap-around services as a key area of support that should be provided [58]. However, the unilateral emphasis on integrated service models may have implications for uptake among PWUD. Past research has demonstrated that previous experiences of stigma and discrimination by PWUD in healthcare settings deterred future uptake of related support services, including community-based services [59–62]. As the MySafe program is intended as a low-barrier model aimed at capturing the hardest-to-reach populations that may not wish or have the resources to access integrated community supports, the findings underscore the importance of adapting the MySafe program to a diversity of settings, including those outside of more medicalized institutions.

In a qualitative study investigating attitudes of PWUD toward embedding buprenorphine treatment at a syringe service program, there were reportedly mixed feelings about the enmeshing of the two related but distinct services [63]. For example, participants reported concerns around changes in service culture due to overcrowding and the perceived institutionalization of low-barrier community supports [63]. Other qualitative findings report similar ambivalence among a sample of PWUD accessing SCS within a community healthcare center with wrap-around supports [62]. For instance, while some participants described benefits of having on-site access to a variety of health and social services, other participants noted barriers related to limited hours of operation, lack of anonymity and privacy, and geographical distances [62]. These barriers were exacerbated by the ongoing criminalization and policing of PWUD which compelled some to avoid services as a means of security [38]. This highlights some of the challenges of embedding harm reduction interventions within existing health services and the potential benefits of standalone services. While efforts are being made toward improving service accessibility, such as the decriminalization of several illicit drugs within BC, advocacy groups argue that threshold quantities fail to realistically reflect drug use quantities of PWUD [64, 65]. While integrated service models may cater to PWUD by providing connections to comprehensive patient care and convenience to those that live within its vicinity, the concentration of services within one area effectively renders PWUD outside of its vicinity without access to such supports. Thus, having multiple MySafe program locations in both housing and community-based organizations would improve access for sub-groups of PWUD with differing willingness and means to access other health and social services.

Moreover, access to the MySafe program would vary considerably depending on each jurisdiction’s existing regulations and clinician buy-in. Participants considered this a significant barrier that manifested into a range of challenges for program uptake such as an inconsistent eligibility criteria and low clinician willingness to onboard participants onto the MySafe program at some sites. Due to Canada’s decentralized healthcare system characterized by the autonomy of provincial governments in allocating funds and delivering healthcare services, a pervasive lack of standardization of drug policy and prescription guidelines has contributed to inequitable access to available supports. This points to the need for a coordinated roll-out of future guidelines across all Colleges paired with robust training of clinicians involved in the delivery of substance use care. Furthermore, provincial mechanisms such as health orders could be enacted to provide security and assurance to clinicians providing access to safer supply in order to safeguard them against repercussions from their respective Colleges. Given the concerns around the inadequacy of hydromorphone, existing and future safer supply programs need to adapt their medication offerings in congruence with the needs of the local context that should include access to diacetylmorphine, including inhalable options [66], as well as stimulant safer supply in the form of extended-release amphetamines, and methylphenidate given the rise of stimulant-involved fatalities in North America [12, 67, 68]. This recommendation is especially pertinent given the influx of benzodiazepine-contaminated drugs into the illicit drug supply in recent times [69].

Our study has several limitations. Firstly, participant views represent a snapshot of the perceived barriers and facilitators primarily at pre-implementation or early implementation stages of the MySafe pilot program. Since some of the pilot locations did not progress toward becoming operational, other influential factors may not be adequately captured by our findings. Secondly, the professional community partners that participated in the study were all involved with the MySafe program in some capacity and may therefore hold a more positive bias on safer supply programs. The views reported in this study may not be representative of professional community partners with more traditional or conservative perspectives on substance use. Future research should focus on the perspectives of a diversity professional community partners to understand potential barriers and facilitators. In addition, the perspectives of PWUD on program design are not captured within this dataset. However, these will be explored in future publications from our larger evaluation of the MySafe program.

## Conclusions

In conclusion, as the overdose crisis evolves with the continually changing unregulated drug supply, the need for additional novel and innovative responses has grown as existing avenues of overdose prevention remain limited and under-utilized. Our findings include valuable insights and recommendations from professional community partners. Community partner-informed research plays an integral role in ensuring program acceptability, implementation, and future rollout of public health interventions, including safer supply programs. Our findings identify current gaps and facilitators underlying the efficacy of one such model, together with perspectives that can be used to inform future policy and best practices in implementing the MySafe program and similar iterations of low-barrier safer supply models across Canada.

## Data Availability

The qualitative datasets for this study are not publicly available given the sensitive nature of the topic, as they contain confidential information that could compromise participant confidentiality and consent.

## Abbreviations

PWUD: People who use drugs
SCS: Supervised consumption services
OAT: Opioid agonist therapy
